# Adaptive Gaze Strategies to Reduce Environmental Uncertainty During a Sequential Visuomotor Behaviour

**DOI:** 10.1038/s41598-018-32504-0

**Published:** 2018-09-20

**Authors:** F. Javier Domínguez-Zamora, Shaila M. Gunn, Daniel S. Marigold

**Affiliations:** 1 Department of Biomedical Physiology and Kinesiology, Simon Fraser University, Burnaby, British Columbia V5A 1S6 Canada; 2 Behavioural and Cognitive Neuroscience Institute, Simon Fraser University, Burnaby, British Columbia V5A 1S6 Canada

## Abstract

People must decide where, when, and for how long to allocate gaze to perform different motor behaviours. However, the factors guiding gaze during these ongoing, natural behaviours are poorly understood. Gaze shifts help acquire information, suggesting that people should direct gaze to locations where environmental details most relevant to the task are uncertain. To explore this, human subjects stepped on a series of targets as they walked. We used different levels of target uncertainty, and through instruction, altered the importance of (or subjective value assigned to) foot-placement accuracy. Gaze time on targets increased with greater target uncertainty when precise foot placement was more important, and these longer gaze times associated with reduced foot-placement error. Gaze times as well as the gaze shifts to and from targets relative to stepping differed depending on the target’s position in the sequence and uncertainty level. Overall, we show that gaze is allocated to reduce uncertainty about target locations, and this depends on the value of this information gain for successful task performance. Furthermore, we show that the spatial-temporal pattern of gaze to resolve uncertainty changes with the evolution of the motor behaviour, indicating a flexible strategy to plan and control movement.

## Introduction

To acquire environmental details necessary for performing a visually guided action, such as locating a landmark, reaching to grasp a glass, avoiding obstacles, and regulating foot placement, appropriate temporal and spatial gaze shifts are required. Consider the situation where you are hiking in the woods; you must identify hazards and obstacles, choose the route you wish to take, and step to desired locations on the ground. Here the decision where, when, and for how long to look has important implications for safety, and thus the coupling between gaze location and foot placement is critical. Although novel stimuli and image salience can capture attention and direct gaze, as supported by computer-based visual tasks and computational models^1^, recent research shows that gaze fixations during more naturalistic behaviours are highly task-relevant^2–9^. For instance, when making a sandwich, eye movements are directed to the knife, the jelly jar, the bread, and the plate before each item is manipulated^2^. When walking across difficult terrain, people predominantly fixate where they will eventually step^5^. However, there is little understanding of how or why task-relevant locations are selected and prioritized, or what determines how much time a location is fixated. Consequently, a central unanswered question emerges: what factors determine how gaze is allocated in visually guided motor behaviours?

Several brain regions implicated in the control of eye movements are sensitive to reward probability^10–13^. For example, the discharge activity of neurons within the monkey lateral intraparietal area (LIP) varies according to the expected (juice) reward associated with an eye movement to a visual target^13^. With walking and other motor actions outside the lab, however, fixating a location does not usually elicit a reward. Rather, gaze shifts help the brain gather relevant details necessary for making a motor decision, such as where to place the foot. Thus, reward alone cannot explain gaze allocation during ongoing, naturalistic behaviours. Interestingly, Foley *et al*.^14^ recently showed that certain LIP neurons change firing rates depending on the expected gain in information needed to perform the second action in a two-step decision task, rather than for the expected reward associated with that subsequent action. This highlights the importance of immediate information gain in shaping action decisions.

Since our knowledge of the world is imperfect, our sensory feedback is noisy, and the environment changes as movement unfolds over time, this means that many environmental features relevant to an action are uncertain. This can affect the execution of movement^15^. To reduce uncertainty and make appropriate motor decisions, we continuously need to acquire new sensory information. Shifts in gaze may facilitate this process^10,16^. However, if gaze shifts serve to reduce uncertainty in the environment, then this likely depends on whether a target of potential interest is relevant (or important) to the task. That is, there is a high value in gaining information from that location. Indeed, the subjective value of a visual stimulus can affect the velocity of saccades^17,18^, and the value assigned to different actions can influence action selection^19^. Sprague and colleagues^20,21^ recently developed a computational model in which a value is assigned to an eye movement by taking account of the expected loss of information if gaze is not directed to a specific location. This model suggests that gaze is allocated to reduce uncertainty if it maximizes a reward associated with accomplishing the goal of the task. Preliminary support for this idea stems from virtual reality-based studies of walking^8^ and driving^7^. For example, Tong *et al*.^8^ found that the number of fixations to a collection of floating objects to avoid increased when their locations were made uncertain by moving them to new random positions. Gaze strategies do not always maximize information gain, though, as some studies on visual search report that people often fixate the most probable rather than the most uncertain target location when under time constraints^22,23^.

In the present study, we ask how uncertainty and the value assigned to an action affects gaze when the sequence of movements associated with the motor behaviour is interdependent; in this case, the step-to-step control of foot placement during walking. We thus extend the predictions of the Sprague *et al*.^20,21^ model to this situation. As such, we test the hypothesis that gaze is allocated to reduce uncertainty about target locations, and that this depends on the value of this gain in information for successful task performance. To accomplish this, subjects performed a visually guided walking paradigm, which required them to step onto three targets while we quantified gaze and foot placement. We used different levels of target uncertainty, and through task instruction, altered the importance of (or subjective value assigned to) foot-placement accuracy. Depending on the experiment, target uncertainties were either consistent or variable within a walking trial. The use of multiple targets and differently structured environments allowed us to also address whether target uncertainty is resolved early in the path and an estimate of the target properties maintained over the course of the sequential action, or is resolved dynamically each step in a flexible manner^14^. We also determined if people gaze at all targets in advance or one step at a time, and whether this changes based on the level of uncertainty or structure of the environment. Although the Sprague *et al*.^20,21^ model does not make predictions for these, given the strong coupling between gaze and stepping in cluttered environments^5,24^, we hypothesized that gaze is allocated to each target in sequence. We further hypothesized increased gaze times for targets early in the sequence in the consistent, but not variable, environment, particularly with greater target uncertainty. Our results show: (1) how uncertainty modulates gaze; (2) how individual gaze fixations relate to the success of the ongoing motor action; and (3) how people adapt their spatial-temporal pattern of gaze for the purposes of planning and control of movement in uncertain environments.

## Methods

### Subjects

Fourteen healthy young adults participated in this study. We excluded two subjects due to problems with the eye tracker, and thus we only analyzed data from twelve subjects (six females and six males; mean age = 24.6 ± 2.6 years). Subjects did not have any known neurological, muscular, or joint disorder that could affect their behaviour in this study but wore corrective lenses if necessary (n = 4). The Office of Research Ethics at Simon Fraser University approved the study, all experiments were performed in accordance with relevant guidelines and regulations, and subjects provided informed written consent prior to participating.

### Experimental Paradigm

**Figure 1:**
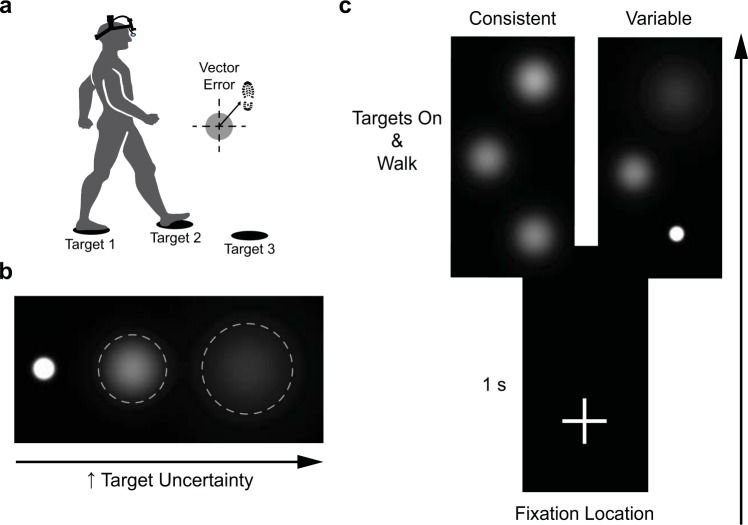
Experimental setup and procedure. (**a**) Visually guided walking paradigm and schematic of vector foot-placement error. (**b**) White two-dimensional Gaussian blob targets for each uncertainty condition. Grey dashed circles show the approximate size of the Medium and High uncertainty targets. These circles are for illustrative purposes only and did not appear during testing. (**c**) Each trial started with subjects fixating a plus sign on the walkway (at a distance of 50 cm with respect to the start position). After one second, we displayed all three targets at random positions and removed the fixation symbol. This signalled to the subjects to start walking. Examples of the Medium uncertainty condition for the consistent environment and one configuration of the variable environment are shown. Person illustration by Amanda Bakkum in (**a**) used with permission.

Subjects performed a visually guided walking paradigm that required them to walk across the lab at a self-selected speed and step onto three irregularly spaced targets without stopping (Fig. 1a). An LCD projector (Epson EX7200) displayed the targets on a black uniform mat covering the walking path. To diminish the effect of environmental references and increase target visibility, subjects walked under reduced light conditions (~0.7 lux, similar to a moonlit night). We configured the targets in MATLAB (The MathWorks, Natick, MA) with the Psychophysics Toolbox, version 3^25,26^. We created three levels of target uncertainty by varying the space constant (i.e., standard deviation) of white, two-dimensional Gaussian blobs: low (σ = 1.6 cm; diameter = 9.5 cm), medium (σ = 5.1 cm; diameter = 30.4 cm), and high (σ = 7.1 cm; diameter = 42.8 cm). Examples of these are shown in Fig. 1b. Thus, with greater standard deviation, there is more uncertainty about the centre of the target; the greater the uncertainty, the more information the subject stands to gain by resolving this uncertainty^27^. This approach is commonly used to manipulate uncertainty of visual targets in studies of sensorimotor control and in psychophysics^28–31^. To confirm that target size does not affect our gaze measures, and thus ensure that any differences we see are the result of target uncertainty, we performed a pilot experiment with six subjects. In this experiment, subjects had to walk and step to the centre of three targets (of similar appearance to the low uncertainty one) in their path. On a trial-to-trial basis, we varied the target diameter; these diameters were similar to those used in the main experiments. The results clearly demonstrated no effects of target size on any of the gaze measures (see Supplementary Figs S1 and S2). Furthermore, target size had no effect on foot-placement error or foot-placement error variability (see Supplementary Fig. S3).

In the main experiments, subjects took one step before and then always stepped with a right-left-right sequence of footfalls across the three targets. We randomized the location of targets on a trial-to-trial basis using nine pre-determined configurations. The mean ± SD distances between targets in the anterior-posterior and medial-lateral directions were 68.8 ± 15.2 cm (range = 42.7 to 85.8 cm) and 27.1 ± 8.7 cm (range = 9.3 to 37.2 cm), respectively. Each trial started with a plus sign to fixate (distance of 50 cm with respect to start position). After 1 s, we displayed all three targets and removed the plus sign (Fig. 1c). This signalled to the subjects to start walking. We instructed subjects to terminate walking after they took one step past the third projected target. We did not provide subjects with feedback on their performance. Subjects were free to look wherever they wanted during the walking trials.

An Optotrak Certus motion capture camera (Northern Digital Inc., Waterloo, Ontario), positioned perpendicular to the walking path, recorded infrared-emitting position markers placed on the head, chest, and bilaterally on each mid-foot (second-third metatarsal head), toe (third metatarsal), and heel at a sampling frequency of 120 Hz. A high-speed mobile eye-tracker (Applied Science Laboratories: model H6-HS, Bedford, MA) mounted on the subject’s head recorded (at 120 Hz) gaze position while walking using the Eye-Head integration feature synchronized with the motion capture camera. We calibrated the eye tracker using the software’s standard 9-point (3-by-3 grid) calibration method at the beginning of the experiments and checked the accuracy periodically throughout testing. Based on the same calibration procedure and seven subjects not involved in this study, the spatial error of the eye tracker in the central (middle calibration point) and periphery (average of the surrounding eight calibration points) is 1.03° ± 0.55° and 1.34° ± 0.36°, respectively.

### Experimental Protocols

Subjects performed two experiments (described below) to study differences in gaze behaviour while walking in consistent and variable environments. We counterbalanced the order across subjects. In each case, we used two tasks in separate blocks of trials that differed in the instructions provided to the subjects. In the Precision-relevant task, we instructed subjects to step with the middle of their foot to the centre of the targets as accurately as possible. In the Precision-irrelevant task, we instructed subjects to step in the targets but told them they did not have to place their foot in the centre. Task instruction served to manipulate the importance of (or subjective value assigned to) foot-placement accuracy. Specifically, the Precision-relevant task instructions increased the value of accurately stepping on the targets. We argue that this makes localizing the target centres (the relevant information) via gaze more critical. This is similar to other work using instruction as means to alter task relevance, or subjective reward of accomplishing a particular action^7,8^. We counterbalanced the order of the Precision-relevant and Precision-irrelevant tasks across subjects in each experiment.

In the consistent environment experiment, all three targets had the same level of uncertainty for a given walking trial. However, we randomly varied the uncertainty levels (low, medium, and high) on a trial-to-trial basis. We presented each uncertainty condition in 15 trials for a total of 45 trials per instruction task (or 90 walking trials overall).

In the variable environment experiment, each of the three targets had a different level of uncertainty (low, medium, and high). We randomized the order of target uncertainty on a trial-to-trial basis. This experiment contained 20 walking trials for the Precision-relevant task and 20 walking trials for the Precision-irrelevant task (for a total of 40 trials).

### Data and Statistical Analyses

To analyze gaze data, we first subtracted head rotation based on position markers attached to the eye tracker from gaze rotation (both in room coordinates) to extract a 3D vector of eye rotation. We then low-pass filtered this data using a 4^th^-order Butterworth algorithm at 15 Hz. We defined saccade onsets and offsets as the times when angular eye rotation exceeded or fell below 100 °/s for a minimum of 16 ms, respectively. Periods >66 ms between a saccade offset and a subsequent saccade onset defined gaze on a particular target or region of the ground. During walking, this means gaze is stabilized on this location but because of the vestibular-ocular reflex the eye is seldom stable in the orbit^8,32^. We used the 30 Hz video provided by a stationary camera and with the gaze location superimposed on the image to verify the presence and location of fixations and to help quantify gaze time on the targets. To assess gaze behaviour, we calculated the following measures, both normalized (i.e., divided) by total walking trial duration to control for any differences in gait speed: the total gaze time on all targets and the average gaze time on a target. Specifically, we used the total time spent looking at all three targets for a given trial in the consistent environment experiment. For the consistent and variable environment experiments, we calculated the average time spent looking at a given target in each trial. Note that for the variable environment experiment, the total gaze time on targets and average gaze time on a target are equivalent for a given trial since each of the three targets has a different level of uncertainty and are treated separately.

To determine how environmental (target) uncertainty affects gaze behaviour while walking, for the consistent experiment, we compared total gaze time on targets between uncertainty conditions and tasks using a two-way (Uncertainty x Task) ANOVA. To determine whether subjects allocate gaze to resolve the target uncertainty differently depending on the target number, in both the consistent and variable environment experiments, we compared average gaze time on a target between uncertainty conditions, tasks, and target number using three-way (Uncertainty x Task x Target) ANOVAs.

We low-pass filtered the kinematic data using a 4^th^-order Butterworth algorithm at 6 Hz. We calculated gait speed using the chest infrared marker as it crossed between the first and third targets. To determine heel contact on the targets, we used the local maximums of the mid-foot vertical velocity profile^33^. To determine toe-off from the targets, we used the local minimums of the anterior-posterior toe marker acceleration profile^34^. We quantified performance by calculating the foot-placement error on each target, defined as the vector distance between the foot (mid-foot infrared marker) and the middle of the target (see Fig. 1a) when the foot’s anterior-posterior velocity and acceleration profiles stabilized to zero. We also calculated foot-placement error variability, defined as the standard deviation of foot-placement error across all three targets in the consistent environment experiment (representing within-trial variability), and the standard deviation of foot-placement error separately for each target in the variable environment experiment (representing between-trial variability since each target had a different level of uncertainty on a given trial).

To determine differences in walking motor performance, we used measures of foot-placement error and error variability as well as gait speed. To determine differences in gait speed, we performed a two-way (Condition x Task) ANOVA and a one-way (Task) ANOVA, for the consistent and variable environment experiments, respectively. We compared foot-placement error between uncertainty conditions, tasks, and target number using three-way (Uncertainty x Task x Target) ANOVAs. We also performed two-way (Uncertainty x Task) ANOVAs with foot-placement error variability. In each ANOVA, gait speed served as a covariate since we found differences between the tasks for each experiment (see *Results* section).

To confirm whether gaze is directed to all targets prior to stepping or each target is dealt with one at a time, we calculated the proportion of gaze shifts from target 1 to target 2 to target 3 in sequence versus not. We then subjected this to a two-way (Uncertainty x Task) ANOVA for the consistent environment experiment and a one-way (Task) ANOVA for the variable environment experiment. We also quantified two spatial-temporal measures of gaze in relation to foot-placement events^24^. Specifically, we calculated the time interval between the end of a saccade to a target and toe-off of the foot about to step to that same target (TO-interval), and the time difference between the onset of a saccade away from a target and heel contact on it (HC-interval). Positive TO-interval values represent gaze shifts to the target after TO, and negative values represent gaze shifts to the target before TO. For the HC-interval measure, positive values represent gaze shifts to a subsequent target after HC, and negative values represent gaze shifts away from the target before HC on it. We used separate three-way (Uncertainty x Task x Target) ANOVAs, with gait speed as a covariate, to determine differences in the mean TO-interval and HC-interval in both the consistent and variable environment experiments.

To identify a relationship between gaze behaviour and action, we performed different mixed-model ANCOVAs. For both the consistent and variable environment experiments, in one model, we used foot-placement error as the dependent variable, with average gaze time on a target as a continuous predictor variable, uncertainty condition as a categorical predictor variable, and subject as a random effect. We performed this analysis separately for each target number because of strong target number effects for each gaze and kinematic measure. In a second set of models for the consistent environment experiment, we used foot-placement error (collapsed across targets) or foot-placement error variability as the dependent variable, total gaze time on targets (summed across targets) as a continuous predictor variable, uncertainty condition as a categorical predictor variable, and subject as a random effect.

We used JMP 12 software (SAS Institute Inc., Cary, NC) with an alpha level of 0.05 for all statistical analyses. For all ANOVAs, we included subject as a random effect, and used Tukey’s post hoc tests when we found significant main effects and/or interactions to determine differences between specific uncertainty conditions or target numbers.

## Results

### Effect of uncertainty on gaze time

**Figure 2:**
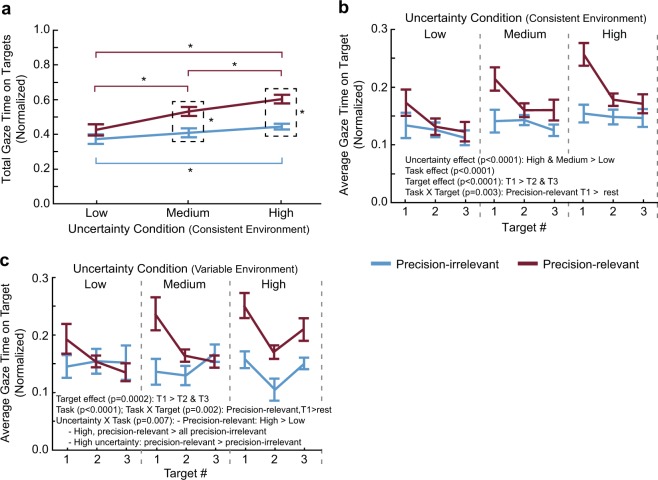
Gaze behaviour. Total gaze time on targets (**a**) and average gaze time on a target (**b**) across uncertainty conditions and tasks in the consistent environment experiment. (**c**) Average gaze time on a target for each uncertainty level and task in the variable environment experiment. Data are represented as mean ± SE. Gaze times are normalized (i.e., divided) by trial duration to control for differences in gait speed between trials and conditions. *Indicates that values are significantly different from each other based on post hoc tests following a significant Task X Uncertainty condition interaction (p < 0.05).

Does target uncertainty influence gaze behaviour? Does it depend on the importance of foot-placement accuracy? We first considered whether subjects allocate a greater amount of time to more uncertain targets, quantified by the total gaze time on targets (summed across all targets and normalized to walking trial duration). As shown in Fig. 2a for the consistent environment, total gaze time increased from the Low to the Medium to the High uncertainty conditions in the Precision-relevant task (Uncertainty X Task interaction: F_2,55_ = 5.4, p = 0.007). In fact, total gaze time increased by 41.6% between the High and Low uncertainty conditions. In the Precision-irrelevant task, subjects spent more time looking at the targets in the High uncertainty condition than in the Low uncertainty condition. However, this represented only a 19.3% increase. We also found an increase of 35.6% and 30.1% total gaze time on targets for both the High and Medium uncertainty conditions, respectively, when the task required a high degree of precision compared to a low degree of precision.

We next considered whether the average gaze time on a target differs based on target uncertainty and importantly, whether this allocation depends on the target number in the stepping sequence and/or task instruction. Figure 2b illustrates the results of this analysis for the consistent environment. We found significant main effects of uncertainty (F_2,187_ = 12.4, p < 0.0001), target number (F_2,187_ = 11.6, p < 0.0001), and task (F_1,187_ = 28.6, p < 0.0001). Specifically, we found increased average gaze time on a target in the High and Medium uncertainty conditions versus the Low uncertainty condition. Gaze on target 1 in the High uncertainty condition, for instance, likely drove this result: average gaze time increased by 48.4% compared to the Low uncertainty condition. Subjects also showed increased average gaze time on target 1 compared to targets 2 and 3, and for the Precision-relevant versus the Precision-irrelevant task. Based on a significant Target x Task interaction (F_2,187_ = 6.0, p = 0.003), subjects had greater average gaze time on target 1 in the Precision-relevant task compared to all other targets in both tasks.

The terrain we encounter when walking (and the uncertainty associated with it) is often variable in nature. In our variable environment experiment, gaze time on targets depended on the task and the target uncertainty (Uncertainty X Task interaction: F_2,184_ = 5.2, p = 0.007). Specifically, in the Precision-relevant task, subjects spent a greater amount of time looking at the High uncertainty target compared to the Low uncertainty target (Fig. 2c). This represented a 32.4% increase when considering target 1. Visual conspicuity (or image salience) cannot explain the increased gaze time since the Low uncertainty targets had the greatest pixel intensity. Subjects also spent more time looking at the High uncertainty target in the Precision-relevant task versus the Precision-irrelevant task. In addition, we found a Target X Task interaction (F_2,184_ = 6.7, p = 0.002), such that subjects had greater gaze time on target 1 in the Precision-relevant task compared to the rest of the targets in both tasks.

Taken together, the results of the consistent and variable environment experiments suggest that uncertainty influences gaze behaviour during visually guided walking but this largely depends on the importance of (or subjective value assigned to) foot-placement accuracy. As evident in the increased gaze times to the first target in the sequence, it also appears that subjects attempt to resolve uncertainty early in the path.

### How does target uncertainty affect motor performance?

In the consistent environment, to accommodate the different uncertainty conditions, subjects did not change their gait speed (F_2,55_ = 0.9, p = 0.417). However, we found that subjects walked slower in the Precision-relevant task (1.04 ± 0.15 m/s) when compared to the Precision-irrelevant task (1.15 ± 0.13 m/s) (F_1,55_ = 25.5, p < 0.0001). In the variable environment, subjects also decreased gait speed in the Precision-relevant task (1.08 ± 0.11 m/s) when compared to the Precision-irrelevant task (1.14 ± 0.12 m/s) (F_1,11_ = 9.7, p = 0.010). Regardless, our gaze measures are normalized to trial duration, thereby ensuring that any minor differences in gait speed were controlled. However, we included gait speed as a covariate for the foot-placement measures.

**Figure 3:**
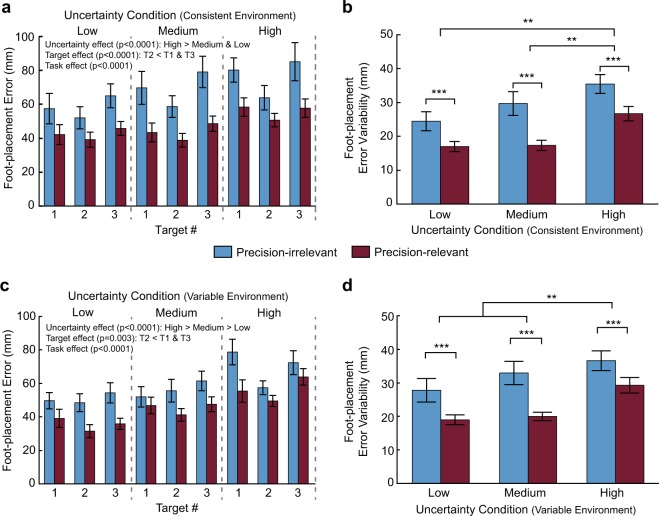
Foot-placement accuracy. Foot-placement error by target for the consistent environment (**a**) and variable environment (**c**) across uncertainty conditions and tasks is shown. Within-trial foot-placement error variability for the consistent environment (**b**) and between-trial foot-placement error variability for the variable environment (**d**) across uncertainty conditions and tasks are shown. Data are represented as mean ± SE. **Indicates uncertainty conditions are significantly different from each other (uncertainty main effect: p < 0.05). ***Indicates tasks are significantly different from each other (task main effect: p < 0.05).

We next determined if the increase in gaze allocation to more uncertain targets in the Precision-relevant task resulted in better foot-placement accuracy. Thus, we first compared foot-placement error between uncertainty conditions, task, and target number (Fig. 3). In the consistent environment, with low target uncertainty, subjects maintained foot-placement error around 40 mm in the Precision-relevant task. We found that subjects had significantly less foot-placement error in the Precision-relevant task compared to the Precision-irrelevant task (F_1,192_ = 37.9, p < 0.0001), as expected (Fig. 3a). This decrease ranged between 16.6% and 38.6% depending on the target and condition. In addition, subjects had significantly greater error in the High uncertainty condition compared to the Medium and Low uncertainty conditions (F_2,186_ = 13.9, p < 0.0001). We subsequently compared foot-placement error variability (i.e., the standard deviation of error across targets within a trial) between uncertainty conditions and tasks. Subjects had greater error variability in the High versus the Medium and Low uncertainty conditions (main effect of uncertainty: F_2,55_ = 20.8, p < 0.0001; Fig. 3b). We also found increased error variability in the Precision-irrelevant task compared to the Precision-relevant task (F_1,59_ = 31.2, p < 0.0001).

In the variable environment, average foot-placement error ranged between 31.5 mm with low uncertainty targets in the Precision-relevant task to 78.7 mm with high uncertainty targets in the Precision-irrelevant task (Fig. 3c). Greater target uncertainty resulted in increased foot-placement error (main effect of uncertainty: F_2,186_ = 29.4, p < 0.0001). Specifically, subjects had larger foot-placement error when stepping on the High uncertainty target compared with both the Medium and Low uncertainty targets, and had larger error with the Medium uncertainty targets compared to the Low uncertainty targets. Subjects also reduced foot-placement error (main effect of task: F_1,188_ = 31.6, p < 0.0001) in the Precision-relevant task compared with the Precision-irrelevant task. We found a similar result for between-trial foot-placement error variability (main effect of task: F_1,65_ = 25.6, p < 0.0001; Fig. 3d). In addition, subjects displayed greater between-trial error variability (main effect of uncertainty: F_2,55_ = 10.0, p = 0.0002) with High uncertainty targets compared to Medium and Low uncertainty targets.

### The spatial-temporal allocation of gaze depends on several factors

**Figure 4:**
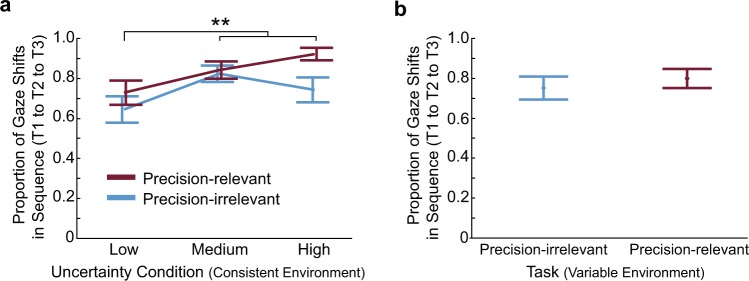
Sequence of gaze shifts. (**a**) Proportion of gaze shifts to sequential targets (T1 to T2 to T3) in the consistent environment with different target uncertainties. (**b**) Proportion of gaze shifts to sequential targets (T1 to T2 to T3) in the variable environment. **Indicates uncertainty conditions are significantly different from each other (uncertainty main effect: p < 0.05).

Is gaze allocated to each target in sequence? Does this depend on target uncertainty? To address these questions, we calculated the proportion of gaze shifts to the targets in sequence. Although all three targets were presented at the same time, and all targets were visible throughout the trial, subjects chose to shift gaze in sequence, from target 1 to target 2 to target 3. This is quantified and shown in Fig. 4. In the consistent environment, the Medium and High uncertainty conditions showed the highest proportion of gaze shifts in this pattern, which differed significantly from the Low uncertainty condition (F_2,55_ = 10.0, p = 0.0002). The proportion of gaze shifts in this pattern was also higher in the Precision-relevant vs. Precision-irrelevant task (F_1,55_ = 9.4, p = 0.003). Regardless of condition or task, the proportion of gaze shifts in this sequence occurred over 63% of trials (with the highest proportion—over 92% of trials—for the High uncertainty condition in the Precision-relevant task). In the variable environment, gaze shifted in sequence in over 75% of trials, with no difference between the Precision-relevant and Precision-irrelevant tasks (F_1,11_ = 0.7, p = 0.427). This occurred regardless of the order of target uncertainty. Interestingly, subjects rarely re-fixated a target once shifting gaze to a new one. In fact, only 0.06 ± 0.08 re-fixations per walking trial occurred across the conditions and tasks in the consistent environment (range: 0.04 to 0.09), and only 0.05 ± 0.07 re-fixations per walking trial occurred across tasks in the variable environment (range: 0.04 to 0.06).

**Figure 5:**
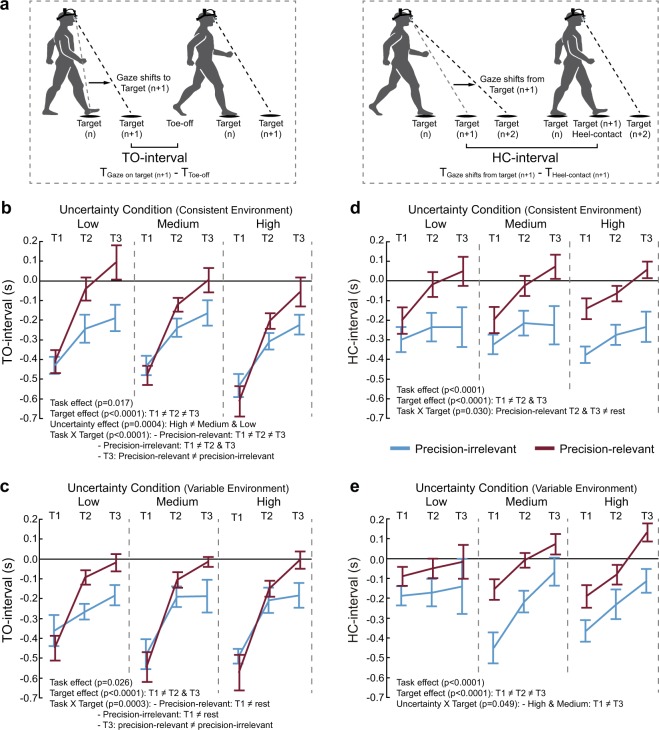
Gaze-foot spatial-temporal coupling. (**a**) The toe-off interval (TO-interval) is the time at which the subject fixates the target minus the time of TO for stepping on it, where negative values indicate a saccade and subsequent fixation made to the target before TO. The heel contact interval (HC-interval) is the time at which a subject saccades away from the stepping target minus the time of HC on that target, where negative values indicate a saccade away before HC on the target. Illustrated are the TO-intervals across uncertainty conditions, tasks, and target number for the consistent (**b**) and the variable (**c**) environments. Also illustrated are the HC-intervals across uncertainty conditions, tasks, and target number for the consistent (**d**) and the variable (**e**) environments. Data are represented as mean ± SE. Person illustrations by Amanda Bakkum in (a) used with permission.

How does target uncertainty affect the spatial-temporal allocation of gaze for the purpose of planning and guiding foot placement in the precision walking paradigm? To address this, we determined the time interval between a saccade to a target and toe-off (TO) of the foot about to step to that same target (Fig. 5a, left panel), as well as the time interval between a saccade away from a target and heel contact (HC) on that same target (Fig. 5a, right panel).

Whereas total gaze time on a target relates to both planning and guiding foot placement, our TO-interval measure reflects primarily visuomotor planning of the upcoming step. In the majority of cases we found negative TO-intervals, indicating that gaze shifted to the target prior to initiating swing phase to step on it, and thus supporting this argument (Fig. 5b,c). One can also consider this as gaze leading the step. In the consistent environment (Fig. 5b), subjects fixated a target sooner before TO of the step towards it in the High uncertainty condition compared to the Medium and Low uncertainty conditions (uncertainty main effect: F_2,187_ = 8.2, p = 0.0004). We also found a significant Target X Task interaction (F_2,186_ = 9.9, p < 0.0001). Post hoc tests indicated that, in the Precision-relevant task, subjects fixated target 1 earlier relative to initiating the step towards it (i.e., a more negative TO-interval) compared to when encountering target 2, which in turn occurred earlier than for target 3. With high target uncertainty, the interval ranged from 608 ms for target 1 to 58 ms for target 3. In the Precision-irrelevant task, subjects shifted gaze earlier to target 1 compared to targets 2 and 3. In both tasks of the variable environment (Fig. 5c), subjects fixated target 1 earlier relative to initiating the step towards it compared to targets 2 and 3 (Task x Target interaction: F_2,184_ = 8.5, p = 0.0003). In contrast to the consistent environment, here we found no effect of uncertainty on TO-intervals (F_2,184_ = 0.8, p = 0.445).

Visual guidance of leg trajectory to ensure accurate foot placement in the precision walking paradigm is primarily reflected in the HC-interval measure. If we examine the consistent environment (Fig. 5d), we find that gaze shifted away from the stepping target ~270 ms prior to footfall on it in the Precision-irrelevant task. In the Precision-relevant task, this amount varied based on the target number in the sequence of footfalls. This is supported by a significant Task X Target interaction (F_2,186_ = 3.6, p = 0.030). Post hoc tests indicated that, in the Precision-relevant task, subjects made a saccade away from the target they were about to step on sooner when encountering target 1 (i.e., more negative HC-interval) compared to targets 2 and 3. However, uncertainty condition did not affect HC-intervals in this environment (F_2,186_ = 0.2, p = 0.786).

In the variable environment (Fig. 5e), we found a significant Condition X Target interaction (F_4,185_ = 2.4, p = 0.049) for the HC-interval. Specifically, in both the High and Medium uncertainty conditions, subjects maintained fixation longer on target 3 relative to stepping on it compared to target 1. In the High uncertainty condition of the Precision-relevant task, for instance, subjects shifted gaze from target 3 ~130 ms after footfall on it but shifted gaze from target 1 ~200 ms prior to footfall on it. We also found a main effect of target (F_2,184_ = 24.3, p < 0.0001), such that the HC-interval differed between all three targets, and a main effect of task (F_1,189_ = 22.4 p < 0.0001), such that subjects maintained fixation on the target they were stepping on longer when precision was more relevant.

Taken together, the timing of gaze shifts to and from targets dynamically changes with each step. This spatial-temporal pattern depends on both the importance of foot-placement accuracy and target uncertainty.

### Gaze times are associated with foot-placement accuracy

**Figure 6:**
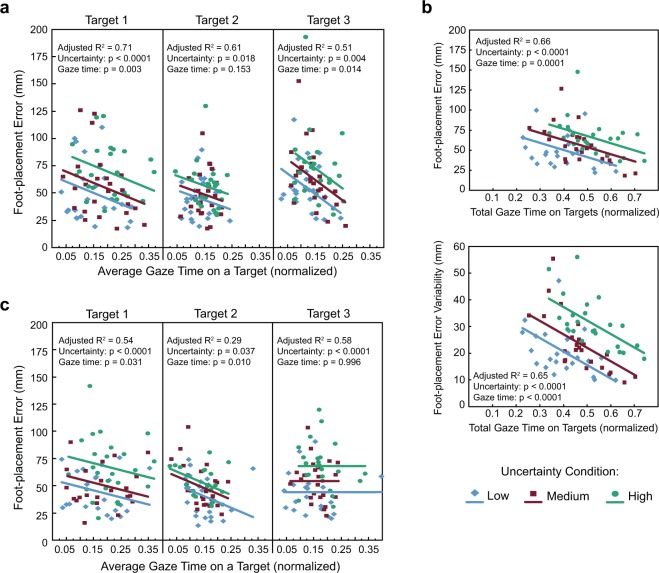
Relationship between gaze behaviour and foot-placement accuracy. (**a**) Scatterplots of average gaze time on a target and foot-placement error for the Low, Medium, and High uncertainty conditions in the consistent environment. (**b**) Scatterplots of total gaze time on targets and foot-placement error (top panel) or foot-placement error variability (bottom panel) for the Low, Medium, and High uncertainty conditions in the consistent environment. (**c**) Scatterplots of average gaze time on a target and foot-placement error for the Low, Medium, and High uncertainty conditions in the variable environment. Solid lines show the linear fits obtained from the ANCOVAs.

To show a relationship between gaze behaviour and foot-placement accuracy in the consistent environment, we performed separate mixed model ANCOVAs. As illustrated in Fig. 6a, increases in average gaze time on a target associated with smaller foot-placement error with respect to target 1 (F_1,64_ = 9.9, p = 0.003) and target 3 (F_1,59_ = 6.4, p = 0.014), though not with target 2 (F_1,68_ = 2.1, p = 0.153). The fit lines between error and gaze time for each uncertainty condition are parallel, with the High uncertainty condition significantly different than the Medium and Low conditions with respect to target 1 (F_2,58_ = 12.7, p < 0.0001), and the High uncertainty condition different than the Low uncertainty condition with respect to target 2 (F_2,59_ = 4.3, p = 0.018) and target 3 (F_2,60_ = 6.0, p = 0.004). This means that the average gaze time on a target needed to reduce foot-placement error to a given value increases with target uncertainty. It also means that if subjects allocate the same average gaze time on target 1 in the Low uncertainty condition as when target 1 has high uncertainty, foot-placement error increases by 24.9 mm; the increase is 23.8 mm with respect to target 3. When we use total gaze time on targets and foot-placement error (averaged across the targets), we observed a similar relationship (Fig. 6b, top panel): increased total gaze time associated with reduced foot-placement error (F_1,67_ = 17.0, p 0.0001). The fit lines show greater error in the High uncertainty condition compared to the Medium and Low uncertainty conditions, and greater error in the Medium uncertainty condition compared to the Low uncertainty condition (F_2,59_ = 13.8, p < 0.0001). We also found that greater total gaze time on targets associated with larger foot-placement error variability (F_1,67_ = 29.3, p < 0.0001; Fig. 6b, bottom panel). The fit lines between error variability and gaze time for each uncertainty condition are parallel, with the High uncertainty condition different than the Medium uncertainty condition, which differed from the Low uncertainty conditions (F_2,60_ = 28.6, p < 0.0001). This means that the total gaze time on targets needed to reduce error variability to a given value increases with greater target uncertainty.

We next determined whether a relationship between gaze behaviour and foot-placement accuracy existed in the variable environment (Fig. 6c). Subjects spent more time looking at the targets to reduce foot-placement error with respect to target 1 (F_1,65_ = 4.9, p = 0.031) and target 2 (F_1,58_ = 7.2, p = 0.010). We did not find this relationship with respect to target 3 (F_1,63_ = 0.0, p = 0.996). The fit lines between error and gaze time differed between the High uncertainty condition and the Medium and Low uncertainty conditions for target 1 (F_2,56_ = 12.9, p < 0.0001) and target 3 (F_2,55_ = 13.4, p < 0.0001), and between the High uncertainty condition and the Low uncertainty condition for target 2 (F_2,57_ = 3.5, p = 0.037).

## Discussion

People must select where, when, and for how long to allocate gaze to perform visually guided motor behaviours. Our findings show that environmental uncertainty affects gaze when having to step accurately during walking. Reduced foot-placement error associated with increased gaze time on targets, and this gaze allocation increased with greater uncertainty as well as when precision was more task-relevant. As such, we propose that shifts in gaze are made to reduce environmental uncertainty for the purpose of improving motor accuracy. This notion may account for a range of observations related to visually guided walking in particular, and visuomotor behaviours in general. In addition, gaze is shifted earlier to and is maintained longer on the first target in the action sequence, suggesting that people may attempt to resolve uncertainty (or acquire information) at the beginning of the path and store an estimate of the target’s properties for subsequent steps. However, gaze shifts towards and away from subsequent targets occur progressively later relative to stepping on each. In fact, in the variable environment, gaze is shifted away from these latter targets after the subject has already made contact with the ground when target uncertainty is greater. We propose that uncertainty is therefore also resolved dynamically on a step-by-step basis in this situation. Taken together, our results suggest a flexibility of gaze patterns to plan and control movement.

### What is the purpose of increasing gaze time on targets?

Gaze time on stepping targets increased with greater target uncertainty and in the precision-relevant task. This increase was not a function of slower gait speed. We propose that the increase in gaze time allows the brain to accumulate evidence of the target position/centre to integrate this information with the state of the body and limbs for the purpose of maximizing stepping accuracy. In other words, it serves to reduce target uncertainty. This is consistent with the model of Sprague and colleagues^20,21^. Importantly, the data in Fig. 6 show a link between gaze time and accuracy. Specifically, our results suggest that to maintain an equivalent degree of foot-placement accuracy, subjects must increase gaze time on a target if that target’s uncertainty is higher. In fact, if subjects adopt a similar gaze time on target 1 in both Low and High uncertainty conditions (Fig. 6a), foot-placement error is increased by ~25 mm in the latter. This is relatively large given that foot-placement error on targets is, on average, normally around 40 mm with no or low target uncertainty. Thus, when target uncertainty is higher, there is a cost related to foot-placement accuracy if gaze time is not increased.

Despite an increase in gaze time on targets, we found greater foot-placement error and error variability in the High uncertainty condition. With several stepping targets present in the path, there is limited time for allocating gaze to any given one. To prolong gaze time on the targets to improve foot-placement accuracy in this condition, subjects would have had to reduce gait speed. However, we found no differences between uncertainty conditions for this measure. Since changes in gait speed increase metabolic cost^35^, it is therefore likely that subjects choose a strategy to minimize this cost yet still maximize accuracy based on the task constraints.

Here we used different instructions to alter the importance of (or subjective value assigned to) foot-placement accuracy. Specifically, the Precision-relevant task instructions increased the value associated with accurately stepping on the targets. According to the model of Sprague and colleagues^20,21^, this increases the importance of reducing target uncertainty for information gain, thus influencing gaze shifts. Furthermore, experiments in monkeys reveal that the choice of saccade target is biased to those associated with a high value for reward^13,36^. In support, we show that gaze times are longer in the Precision-relevant task compared to the Precision-irrelevant task, particularly when target uncertainty is greater. In virtual reality experiments, Sullivan *et al*.^7^ and Tong *et al*.^8^ varied task instruction as a means to manipulate the priority of two or more competing task demands, and found this affected the fixation frequency to objects and other locations. Our results extend the notion that high value stimuli influence gaze to a visually guided walking task where accurate, sequential foot placement is necessary.

We suggest that the increase in gaze time is also driven by a desire to maximize a reward associated with being accurate^10,20,37^. Since we did not provide an actual reward (such as food or money), our argument requires two assumptions: accurate stepping is subjectively rewarding, and regions involved in reward processing are active in its absence. Accurate foot placement is necessary to avoid dangerous hazards in one’s path and prevent falls, thereby ensuring one safely reaches their intended destination or accomplishes a task essential to survival, such as to obtain food. This suggests that the importance of precise foot placement is likely engrained early in life. It is also worth considering that society places of a high value on accuracy; this is reflected in the large monetary rewards given to high-performance athletes. In addition, the human ventral striatum—a region involved in processing reward—is also active in the absence of an actual reward when feedback of performance in a visuomotor task is available^38^. Furthermore, the activity of this region increases following correct versus incorrect responses in a target-matching task despite no reward and no feedback^37^. In the Sprague *et al*.^20^ model, a simulated agent walks and performs several motor behaviours, including avoiding obstacles and picking up litter. It uses a standard reinforcement learning algorithm to determine which action the agent should take for each motor behaviour given the current state of the agent and environment, with the goal of maximizing expected reward across all behaviours. Gaze is allocated based on its ability to reduce environmental uncertainty where it stands to provide the largest gain in information (or there is the greatest risk of losing a reward). Ultimately, this model argues that gaze shifts act to ensure a motor behaviour is accomplished and reward is maximized, and our results support many of its core assumptions.

### The influence of sequential target-footfalls on gaze behaviour

The negative TO-intervals suggest that people acquire relevant information to plan gait modifications before initiating limb movement. This is similar to earlier work where subjects had to step onto non-sequential targets while walking^24^, and supports the idea that gaze leads or anticipates locomotor trajectory^39,40^. These results are also consistent with recent research that demonstrates a critical phase of the gait cycle prior to toe-off during which visual feedback regarding the future footfall target is particularly important^41–43^. These authors argue that this allows the nervous system to exploit at least one determinant of the passive trajectory of the body’s centre of mass to maximize the energetic efficiency of walking across complex terrain. However, this work also suggests that people do not rely on visual feedback of the target during swing phase. In contrast, we show that subjects maintain gaze on the target well into this phase of the gait cycle, which is reflected in HC-intervals close to or greater than zero. This implies continued importance of visual feedback, which is most apparent in the higher uncertainty conditions of the Precision-relevant task. These discrepancies may relate to at least two methodological differences; we recorded gaze behaviour and used different target uncertainties, whereas Matthis and colleagues^41–43^ changed when and where similar targets were visible.

In the vast majority of trials in both experiments, subjects allocated gaze to each target in sequence despite the fact that all targets were visible. Importantly, how our subjects allocated gaze to identify the target position to plan and control foot placement changed over the sequence of target-footfalls. Gaze shifts happened earlier and gaze times were longer in relation to the first target compared to the others, especially with greater target uncertainty. A progressive shift towards maintaining gaze longer on later targets relative to stepping on them, particularly in the Precision-relevant task where accuracy is paramount, accompanied these changes.

Why does this sequential target effect occur? One explanation is that subjects have more time to deal with the first target due to the extra footfall before stepping on it. The same logic may explain the HC-intervals for target 3 that show subjects shifted gaze away from this target later than the others when no additional targets in the path were present. If this were the case, however, we should not see differences in gaze times on target 1 for the Precision-relevant and Precision-irrelevant tasks like we did. Nor should we have found an effect of task and uncertainty on HC-intervals. Subjects were also free to look at any target during this period of time, but we found that in the majority of trials they shifted gaze to each target in sequence as they approached them. Thus, this explanation cannot entirely account for these results.

We propose that people attempt to resolve target uncertainty early in the sequence of steps, and then use this accumulation of evidence for planning foot placement on subsequent targets. This implies that people form an internal model of the properties of the targets, with an estimate of the target centre carried forward to future targets, and hence the reduction in gaze times. The visual system is indeed capable of learning the statistical structure of the environment and objects within it^44–46^. Furthermore, the brain can combine this prior estimate with new visual input based on the uncertainty of the information to guide both eye movements^45^ and foot placement^15^. This strategy makes sense when target uncertainty is consistent across the environment. However, this strategy may be less efficient in a variable environment, where the uncertainty of one target does not necessarily relate to the uncertainty of subsequent targets, particularly if their uncertainty is higher. Although we found a strong target effect on gaze times similar to the consistent environment, gaze time increased again when the high uncertainty target was in the third position (Fig. 2c). In this case, we observed prolonged gaze on the high uncertainty target relative to footfall (Fig. 5e). Thus, when to shift gaze to and from a target represents another strategy—in addition to manipulating the duration of gaze—to deal with target uncertainty. Collectively, our results suggest a flexibility of gaze patterns and the ability to dynamically reduce uncertainty on a step-by-step basis. They also highlight the importance of studying complex, sequential motor behaviours typical of everyday life in addition to discrete saccade and reaching tasks, and may indicate a need for revising existing computational models of gaze behaviour for motor control^20,21^.

### Reducing environmental uncertainty may explain certain aspects of visual sampling behaviour during walking

Our results may help to explain a seemingly disparate collection of findings associated with visually guided walking over the past several decades. For instance, if subjects are given control over the amount of visual input they receive, the frequency and duration of visual sampling increases with more complex terrain^47^. Furthermore, as terrain irregularity or complexity increases, people direct gaze closer to their feet^5,32,48,49^. Although the desire to avoid tripping or falling could explain this behaviour, our data suggest that the reason relates to uncertainty in terrain characteristics, something previously proposed^49^ but not formally tested. When walking through crowds or passing through sliding doors, fixation duration and frequency to these objects increase as their motion or trajectory becomes more unpredictable^45,50^. Additionally, people with reduced peripheral vision direct gaze to a wider area of the environment compared to normally-sighted controls^51^. Finally, when the lower visual field is blocked, people pitch their head downward to a greater extent to compensate^52^. Overall, we suggest that one can interpret each of these gaze or behavioural strategies as seeking to reduce uncertainty (or gain relevant information) about the environment to achieve a goal. Together with our results, this suggests that people can flexibly adjust their visual sampling based on task constraints.

## Electronic supplementary material

Supplementary figures

## Data Availability

The analysed data from the current study are available from the corresponding author on reasonable request.
