# Supplementary material for: Adaptive Gaze Strategies to Reduce Environmental Uncertainty During a Sequential Visuomotor Behaviour
Source: Sci Rep. 2018 Sep 20;8:14112. doi: 10.1038/s41598-018-32504-0 (PMC6148321; doi:10.1038/s41598-018-32504-0)
Supplement: Supplementary file 1 — Supplementary figures [file 41598_2018_32504_MOESM1_ESM.pdf]

Supplementary Information

Adaptive gaze strategies to reduce environmental uncertainty during a sequential visuomotor behaviour

F. Javier Domínguez-Zamora, Shaila M. Gunn, and Daniel S. Marigold

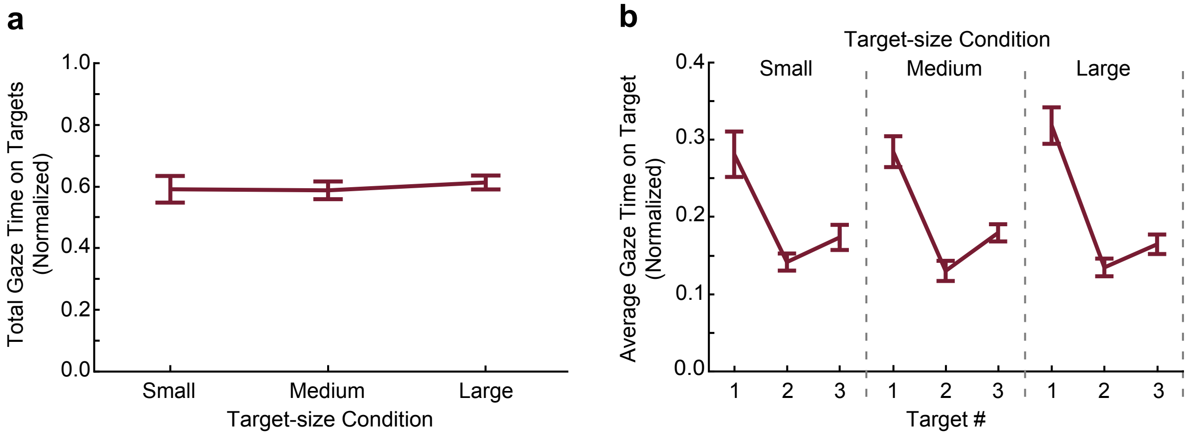

Supplementary Figure S1: Target size does not affect gaze times. In the pilot experiment, we used targets that were similar in appearance to the low uncertainty target of the main experiments. All three targets in a given walking trial were of the same diameter. The diameters of the small, medium, and large targets were 10.5 cm, 30.5 cm, and 41.5 cm, respectively. We presented these target-size conditions (5 walking trials each) in random order. Instructions to subjects matched that for the precision-relevant task in the main experiments. See the main article for a description of the gaze measures. **(a)** For total gaze times, we found no significant main effect of target size ($F_{2,10} = 0.56$, $p = 0.590$). **(b)** For average gaze times, we found no significant main effect of target size ($F_{2,40} = 0.23$, $p = 0.793$) or target size x target # interaction ($F_{4,40} = 0.95$, $p = 0.447$). However, we found that average gaze times to each target differed (target # main effect: $F_{2,40} = 86.8$, $p < 0.0001$), with average gaze time to target 1 greater than the other targets and average gaze time to target 3 greater than target 2. Gaze times are slightly larger in this pilot experiment compared to the main experiments due to the fact that subjects took an extra step before the first target and thus had more time to look at it.

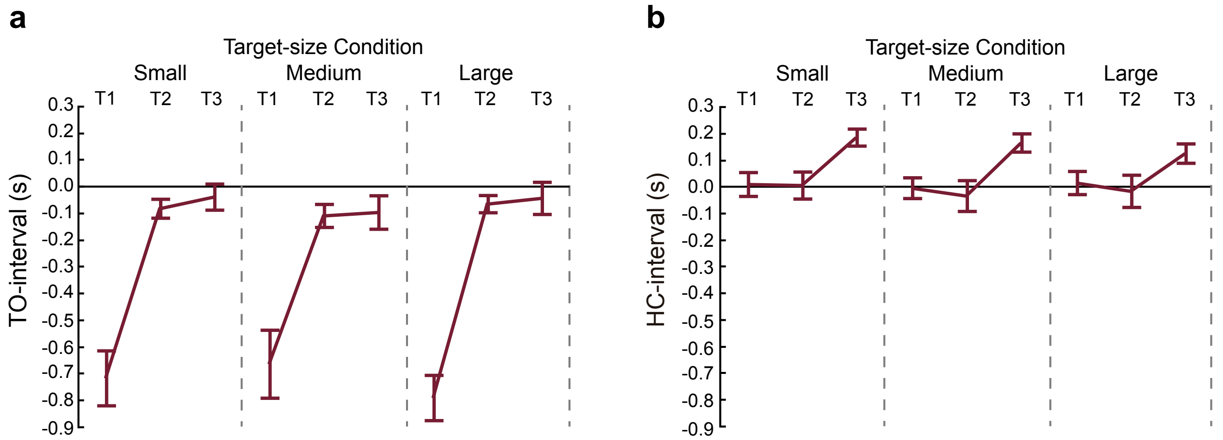

Supplementary Figure S2: Target size does not affect gaze-foot intervals. In the pilot experiment, we used targets that were similar in appearance to the low uncertainty target of the main experiments. All three targets in a given walking trial were of the same diameter. The diameters of the small, medium, and large targets were 10.5 cm, 30.5 cm, and 41.5 cm, respectively. We presented these target-size conditions (5 walking trials each) in random order. Instructions to subjects matched that for the precision-relevant task in the main experiments. See the main article for a description of the gaze measures and Fig. 5A for illustrations. Here we also used gait speed as a covariate. **(a)** For the TO-interval, we found no significant main effect of target size ($F_{2,41} = 0.04$, $p = 0.959$) or target size x target # interaction ($F_{4,39} = 1.01$, $p = 0.412$). However, we found that TO-intervals to each target differed (target # main effect: $F_{2,39} = 164.6$, $p < 0.0001$), where TO-interval to target 1 (T1) differed from TO-intervals to target 2 (T2) and target 3 (T3). The TO-intervals for T1 are slightly larger in this pilot experiment compared to the main experiments due to the fact that subjects took an extra step before the first target and thus had more time to look at it. **(b)** For the HC-interval, we found no significant main effect of target size ($F_{2,42} = 0.02$, $p = 0.979$) or target size x target # interaction ($F_{4,39} = 0.72$, $p = 0.585$). However, we found a target # main effect ($F_{2,39} = 48.3$, $p < 0.0001$), where the HC-interval to T3 differed from the HC-intervals to T2 and T1.

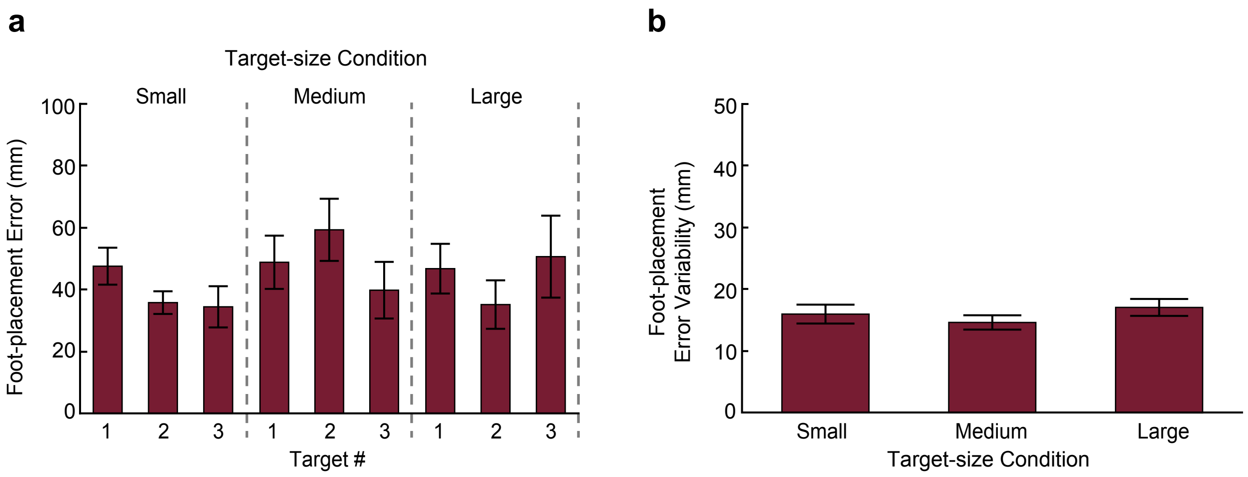

Supplementary Figure S3: Target size does not affect foot-placement accuracy. In the pilot experiment, we used targets that were similar in appearance to the low uncertainty target of the main experiments. All three targets in a given walking trial were of the same diameter. The diameters of the small, medium, and large targets were 10.5 cm, 30.5 cm, and 41.5 cm, respectively. We presented these target-size conditions (5 walking trials each) in random order. Instructions to subjects matched that for the precision-relevant task in the main experiments. See the main article for a description of the foot-placement error measures. Here we also used gait speed as a covariate in the analysis. **(a)** For foot-placement error, we found no significant main effect of target size ($F_{2,41}$ = 1.4, p = 0.257) or target # ($F_{2,39}$ = 1.7, p = 0.196). However, we found a significant target size x target # interaction ($F_{4,39}$ = 5.3, p = 0.002). Tukey post hoc tests only showed greater error on target 2 in the medium target-size condition compared to error on target 2 in the large target-size condition and target 3 in the medium target-size condition. **(b)** For foot-placement error variability, we found no significant main effect of target size ($F_{2,11}$ = 1.1, p = 0.363). Overall, the results suggest no consistent effect of target size on foot-placement error or error variability.
